# Modeling Expected Shortfall Using Tail Entropy

**DOI:** 10.3390/e21121204

**Published:** 2019-12-07

**Authors:** Daniel Traian Pele, Emese Lazar, Miruna Mazurencu-Marinescu-Pele

**Affiliations:** 1 Department of Statistics and Econometrics, Faculty of Cybernetics, Statistics and Economic Informatics, The Bucharest University of Economic Studies, Piata Romana, nr.6, Sector 1, 010371 Bucharest, Romania; miruna@ase.ro; 2 Henley Business School, University of Reading, ICMA Centre, Whiteknights, Reading RG6 6BA, UK; e.lazar@icmacentre.ac.uk

**Keywords:** expected shortfall, measure of risk, information entropy, tail risk, C14, C22, G10

## Abstract

Given the recent replacement of value-at-risk as the regulatory standard measure of risk with expected shortfall (ES) undertaken by the Basel Committee on Banking Supervision, it is imperative that ES gives correct estimates for the value of expected levels of losses in crisis situations. However, the measurement of ES is affected by a lack of observations in the tail of the distribution. While kernel-based smoothing techniques can be used to partially circumvent this problem, in this paper we propose a simple nonparametric tail measure of risk based on information entropy and compare its backtesting performance with that of other standard ES models.

## 1. Introduction

Past banking regulation has concerned and current banking regulation concerns the measurement of risk needed for capital calculation, which can be computed based on various models. Additionally, banks and financial institutions need to estimate their own exposure to different relevant risk factors in order to understand the overall riskiness of their activities and to help them prepare for undesirable situations. While value-at-risk (VaR) is the most popular risk measure and has been widely used in the past, it is not a subadditive measure (Artzner et al. [1,2]) and it is a misleading measure for portfolio optimizers [3]. Expected shortfall (ES), which has been popularized by Artzner et al. [1,2], having the advantage of being a coherent measure [4], has increased in importance since the Basel Committee on Banking Supervision ([5,6]) began to bases its regulatory capital calculations on ES estimates at the 2.5% significance level.

The standard way to estimate ES at level α is to simply compute the weighted average of the historical returns in the α-tail of the returns’ distribution, where the weights are the probabilities associated with the returns estimated from historical data (nonparametric method). However, this often gives an estimate that is inaccurate (see McNeil et al. [7]), data-sensitive (due to the low number of observations in the tail), and often characterized by an undesirably large variability.

A comprehensive presentation of the modern techniques used in risk management, especially with regard to estimating value-at-risk, expected shortfall, and spectral risk measures, can be found in Guégan et al. [8].

Several alternatives exist to computing ES, and a comprehensive review can be found in Nadaraj et al. [9]. Starting with semiparametric methods, a strand of this approach is based on extreme value theory, as in McNeil and Frey [10], who apply the theoretical framework of Embrechts et al. [11] and employ the generalized Pareto distribution combined with an ARMA-GARCH(1,1) process. In a later paper, Embrechts et al. [12] propose an ES estimation method based on the Hill estimator. Another semiparametric ES estimation method, using expectiles, has been proposed by Taylor [13] based on an asymmetric regression methodology of Aigner et al. [14] and the CaViaR model of Engle [15]. A smoothing technique, detailed in Schnabel and Eilers [16] and based on the algorithm of Schall [17], can be employed to improve the performance of the ES estimate. Daouia et al. [18] consider expectile-based ES estimation for extreme percentiles and study their asymptotic behavior.

There are many parametric methods used to estimate ES. Nadaraj et al. [9,19] enlist more than 100 approaches, including methods that make different distributional assumptions (Gaussian, Johnson family, Student’s *t*, stable distributions, mixture distributions, generalized Pareto and elliptical distributions, etc.)—see also Broda and Paolella [20]. Many methods employ different econometric formulations (like GARCH, as in a recent paper by Krause and Paolella [21], or ARMA, or even Bayesian approaches); these methods are mostly applied to historical data, but they can be applied within a Monte Carlo simulation setup as well. All these parametric approaches make an assumption of a particular model, so they bear significant model risk.

Chen [22] argues that semiparametric or parametric tail-risk estimation models struggle to specify an adequate model for the tail distribution (due to a lack of data), meaning nonparametric models are preferred. In terms of nonparametric ES estimation (which has the advantage of not employing a particular model and hence not being affected by misspecification risk), in addition to the classical historical ES estimation (which is considered the gold standard), the estimator of Yamai and Yoshiba [3], the estimator of Inui and Kijma [23], the ES estimator of Chen [22], and the methodology of Jadhav et al. [24], which has been shown to perform better than an historical estimator, have all been proposed. Kernel methods based on a kernel-smoothed estimator, as in Scaillet [25], can be used as well, but as shown by Chen [22], smoothing negatively affects the accuracy of ES estimates as it introduces a bias and does not reduce the variance of the estimates. To correct this, Alemany et al. [26] have shown that a double transformed kernel estimator can, in some cases, be a good alternative. Cai and Wang [27] suggest a nonparametric ES estimator using weighted double kernels and study its finite sample performance. The trimmed kernel method of Hill [28] is a bias-corrected estimator with a small error only. Another alternative is provided by the method of Richardson [29] and the algorithm of Fan et al. [30], which reduce the bias but not the error of the estimation (see Inui and Kijma [23]). A two-stage estimation method based on extreme value theory has been proposed by Martins-Filho et al. [31]. In this paper we propose a nonparametric estimator based on tail entropy (TE) and compare it with alternatives.

As entropy is a measure of uncertainty, which is in some ways similar to volatility, it is widely used in physics and the social sciences. In general, the higher the uncertainty of a system, the larger its entropy. The central theme of this paper is risk, which is directly linked to the tail of the distribution of returns (as opposed to its full distribution), so it makes sense to compute entropy based solely on returns that determine risk (that is, the tail of the distribution), which then gives tail entropy, as discussed in detail in this paper. Roughly speaking, tail entropy is defined as the weighted sum of log probabilities in the tail, where the weights are the conditional tail probabilities themselves.

Furthermore, it has to be noted that there is a strong similarity between definitions of ES and tail entropy. While ES computes the weighted average of tail returns, tail entropy calculates the weighted average of the log probabilities of the returns (again, in the tail of the distribution), but the two calculations use the same weights (probabilities). As such, tail entropy is an estimate of tail risk, which is free of the main problem with ES, namely, its direct dependence on the actual observations in the tail, hence the motivation of our paper. Our aim was to study how tail entropy can be used as an efficient measure of risk and how it compares with alternative estimates of ES.

It is natural to compare the theoretical and empirical properties of entropy with those of variance or volatility, with both being measures of uncertainty. While volatility is often considered a measure of risk in finance, here we consider it a measure of uncertainty. To differentiate between the two types of measures, i.e., measures of risk and measures of uncertainty, measures that are symmetric by nature are measures of uncertainty, and (asymmetric) tail measures are considered measures of risk. Thus, tail entropy is a measure of risk. Philippatos and Wilson in 1972 [32], and, later, Ebrahimi et al. [33] suggested that entropy compares favorably to volatility/variance as a measure of uncertainty. The same has been shown by Dionisio et al. [34], with the authors arguing that while variance measures the concentration around the mean, entropy measures the dispersion of the density irrespective of the location of the concentration (also see Ebrahimi et al. [35] and Allen et al. [36]). Moreover, the entropy of a distribution function is strongly related to its tails, as shown by Pele et al. [37], which becomes an important feature for distributions with heavy tails or with an infinite second-order moment for which an estimator of variance is obsolete. Liu et al. [38] compare an entropy-based measure with the classical coefficient of correlation and conclude that their measure has certain superior characteristics as a descriptor of the relationship between time series when compared with the correlation measure.

The study of the relationship between entropy and financial markets goes back more than a decade. Entropy has been used as a measure of stock market efficiency in Zunino et al. [39], while Risso [40] links it to stock market crashes, arguing that the probability of having a crash increases as entropy decreases. Oh et al. [41] use entropy as a measure of the relative efficiency of the foreign exchange (FX) markets. Based on generalized entropy theory, Wang et al. [42] analyze the interactions among agents of stock markets. Entropy has also been used as a tool for the predictability of stock market returns. Maasoumi and Racine [43] show that entropy can capture nonlinear dependence between financial returns. An effective early warning indicator for crisis situations of banking systems has been built by Billio et al. [44]; this indicator considers different definitions of entropy.

Entropy has also been used in option pricing (Stutzer [45] and Stutzer and Kitamura [46]). An application of entropy-based risk measures for decision-making can be found in Yang and Qiu [47]. Applications of Tsallis entropy in risk management can be found in Gradojevic and Gencay [48], Gencay and Gradojevic [49], and Gradojevic and Caric [50]. Bowden [51] introduces directional entropy and uses it to improve the performance of VaR in capturing regime changes. Portfolio optimization based on maximum entropy has been discussed by Geman et al. [52].

The main objectives of this paper were to introduce a measure for expected shortfalls based on tail entropy, to study this measure’s properties, and to compare it with alternative measures of ES. The main advantage of the measure we propose is that it is less sensitive to the actual values of observations in the tail of the distribution (compared to historical ES), making it a more stable measure of tail risk. We use several ES backtests to verify the accuracy of the proposed measure. The rest of the paper is organized as follows. Section 2 derives the theoretical results specifying the steps in estimating tail entropy as a measure of market risk and details our methodology, Section 3 presents an empirical application, and Section 4 concludes.

## 2. Methodology

For a series of asset prices denoted by *S_t_*, in the following we compute and denote log-returns by *X* with realizations ${\left(X_{t}\right)}_{t\in \mathbb{N}}$, i.e.,

(1) $$X_{t+1}=\log\left(S_{t+1}/S_{t}\right).$$

### 2.1. Measures of Risk: VaR and ES

Given the returns series *X*, with *F* denoting its cumulative density (or distribution) function, for an α∈(0,1) the VaR at level *α* is the smallest number with cumulative density at least as big as α, i.e.,

(2) $$VaR_{\alpha }\left(X\right)=-\inf\left\{x|F\left(x\right)\geq \alpha \right\}.$$

In probabilistic terms we can write that

(3) $$P\left(X<-VaR_{\alpha }\left(X\right)\right)=\alpha .$$

In addition, VaR is the negative of the quantile, i.e.,

(4) $$VaR_{\alpha }\left(X\right)=-F_{\alpha }^{-1}.$$

The ES at significance level *α* is the average of the returns below the VaR at level *α*, as given by

(5) $$ES_{\alpha }\left(X\right)=-E\left[x|x\leq -VaR_{\alpha }\left(X\right)\right]=\frac{1}{\alpha }\int_{0}^{\alpha }VaR_{\gamma }\left(X\right)d\gamma .$$

For a continuous variable with density function *f*, the above definition for ES can be rewritten as

(6) $$ES_{\alpha }\left(X\right)=-\frac{1}{\alpha }\left(\int_{-\infty }^{-x_{\alpha }}xf\left(x\right)dx+x_{\alpha }\left(\alpha -P\left[X\leq x_{\alpha }\right]\right)\right),$$

where $x_{\alpha }=\inf\left\{x\in R|P(X\leq x)\geq \alpha \right\}$ is the left α-quantile.

### 2.2. Entropy

Entropy is a measure of uncertainty, which is in some ways similar to volatility. Various definitions exist (e.g., Shannon entropy, Tsallis entropy, and Kullback Cross entropy etc.) based on the informational content of a discrete or continuous random variable (see Zhou et al. [53] for a comprehensive review of entropy measures used in finance). Shannon information entropy is the most commonly used definition of entropy; it quantifies the expected value of the information contained in a discrete distribution.

**Definition** **1 (Shannon information entropy).** *For X, a discrete random variable with probability distribution $X:\left(\begin{array}{l} x_{1}......x_{n}\\ p_{1}......p_{n} \end{array}\right)$, with $p_{i}=P(X=x_{i})$, $0\leq p_{i}\leq 1$, and $\sum_{i}p_{i}=1$, the Shannon information entropy is defined as*

(7) $$H(X)=-\sum_{i}p_{i}\log_{2}p_{i}.$$

For a discrete distribution, the entropy reaches its maximum value of $H(X)=\log_{2}n$ for the uniform distribution when all the p_i_ values are the same (i.e., when there is a high level of uncertainty). Similarly, the entropy will reach its minimum value of 0 for a distribution with zero uncertainty (i.e., one of the probabilities p_i_ is 1 and the rest are all 0). 

For X, a continuous random variable with probability density function f(x), the differential entropy is given by

(8) $$H(f)=-\int_{A}f(x)\log_{2}f(x)dx,\;A=\mathrm{supp}\left(X\right).$$

However, unlike with Shannon entropy, differential entropy does not possess certain desirable properties like invariance to linear transformations and non-negativity (Lorentz [54] and Pele [55]). A measure of entropy similar to Shannon entropy can be defined via a transformation called quantization, as defined below [54].

**Definition** **2 (sampled function).** For f:I=[a,b]→R, a real valued continuous function, $n\in N^{*}$, a fixed number, and $x_{i}=a+(i+1/2)h$, i=0,…n−1, where h=(b−a)/n, the sampled function for f is given by (point sampling)

(9) $$S_{n}(f)(i)=f(x_{i}),\;\;\mathrm{for}\;i=0,\ldots n-1.$$

*For*f:I=[a,b]→R, *which is essentially bounded, the sampled function is (mean sampling)*

(10) $$S_{n}(f)(i)=h^{-1}\int_{x_{i}-h/2}^{x_{i}+h/2}f(x)dx\;\mathrm{for}\;i=0,\ldots n-1.$$

**Definition** **3 (quantization).** *The quantization of a function creates a simple function that approximates the original one. Given q > 0, a quantum, the following function defines a quantization of*f.

(11) $$Q_{q}(f)(x)=(i+1/2)q,\;\mathrm{if}\;f(x)\in [iq,(i+1)q).$$

**Definition** **4 (entropy of a function at quantization level *q*).** *Let f be a measurable and essentially bounded real valued function defined on [a,b] and let q > 0. Let $I_{i}=[iq,(i+1)q)$ and $B_{i}=f^{-1}(I_{i})$. Then, the entropy of f at quantization level q is given by*

(12) $$H_{q}(f)=-\sum_{i}\mu (B_{i})\log_{2}(\mu (B_{i})),$$

*where μ denotes the Lebesgue measure*.

Lorentz’s theorem given below calculates the entropy of a continuous function on a compact interval. Eventually, it helps define the entropy of a probability distribution function for a continuous random variable on a compact interval, regardless of the sampling and quantization.

**Theorem** **(Lorentz [54]).** *Let f be continuous for point sampling, measurable, and essentially bounded for mean sampling. The sampling spacing is 1/n. Let $S_{n}(f)$ be the corresponding sampling; fix q > 0 and let $Q_{q}S_{n}$ be the quantization of the samples with resolution q as in Definition 3. The number of occurrences of (i+1/2)q in $Q_{q}S_{n}$ is $c_{n}(i)=card\{(i+1/2)q\in Q_{q}S_{n}\}$ and the relative probability of the occurrence of the value i is denoted by $p_{n}(i)=\frac{c_{n}(i)}{\sum_{j}c_{n}(j)}=\frac{c_{n}(i)}{n}$. Then, we have*

(13) $$\underset{n\to \infty }{\lim}-\sum_{i}p_{n}(i)\log_{2}p_{n}(i)=H_{q}(f).$$

In the following we assume that we are dealing with a continuous random variable *X* whose support is the set *A* with the distribution function F:R→[0,1], F(x)=P(X<x). If the distribution function is absolutely continuous, then there exists a non-negative, Lebesgue integrable function *f* which is a probability density function (PDF), i.e.,

(i) $F(b)-F(a)=P(a<X<b)=\int_{a}^{b}f(x)dx$;
(ii) $\int_{A}f(x)dx=1$;
(iii) $F(x)=\int_{-\infty }^{x}f(t)dt$.

Assuming that the probability density function *f* is essentially bounded, we can define the entropy of the probability density function *H_q_*(*f*) at the quantization level *q* > 0.

**Definition** **5 (entropy of a probability density function at quantization level *q*).** *For a probability density function f:R→[0,∞) which is essentially bounded, let $I_{i}=[iq,(i+1)q)$ disjoint giving $\underset{i}{\cup }I_{i}=[0,1]$ and $B_{i}=f^{-1}(I_{i})$ not necessarily disjoint, with $\underset{i}{\cup }B_{i}=[0,1]$. Then, the entropy of f at quantization level q is $H_{q}(f)=-\sum_{i}\mu (B_{i})\log_{2}(\mu (B_{i}))$, where μ is the Lebesgue measure.*

Of note is that in general, for a probability density function defined on set A not necessarily of finite measure, it is possible to consider this function’s restriction on a compact interval, i.e.,

f|[a,b]:[a,b]→Im(f|[a,b]), f|[a,b](x)=f(x).

Then, f|[a,b]:[a,b]→Im(f|[a,b]) satisfies the conditions of Lorentz’s theorem, meaning the entropy can be defined.

The above framework can be applied to estimation of the entropy of a probability density function of a continuous random variable *X.*

Next, we present the estimation algorithm of the entropy of a probability density function. Let $X_{0},....,X_{n-1}$ be a sample of an *i.i.d.* variable with probability density function *f*. The Algorithm 1 (see below) estimates the entropy of a probability density function (see Pele et al. [37]).
**Algorithm 1. Estimation of the entropy of a probability density function.**Estimate the probability density function, obtaining values ${\hat{f}}_{n}(X_{i})$ for i=0,…n−1;Sample from the probability density function using the sampled function $S_{n}({\hat{f}}_{n})(i)={\hat{f}}_{n}(X_{i})$ for i=0,…n−1;Define a quantum q>0; then $Q_{q}S_{n}({\hat{f}}_{n})(j)=(i+1/2)q$, if ${\hat{f}}_{n}(X_{j})\in [iq,(i+1)q)$;Compute the probabilities $p_{n}(i)=\frac{c_{n}(i)}{\sum_{j}c_{n}(j)}=\frac{c_{n}(i)}{n}=\frac{card\{{\hat{f}}_{n}(X_{j})\in [iq,(i+1)q)\}}{n}$;Estimate the entropy of the probability density function, i.e., $H_{q}({\hat{f}}_{n})=-\sum_{i}p_{n}(i)\log_{2}p_{n}(i)$

A similar approach can be found in Miśkiewicz [56], where two possible algorithms for estimating entropy of a continuous probability density function are discussed.

The entropy of the probability density function reaches its maximum value for the uniform distribution. A dimensionless measure of uncertainty, normalized entropy, can be defined as the ratio between the entropy of the probability density function and the entropy of the uniform distribution, i.e.,

(14) $$NH_{q}({\hat{f}}_{n})=\frac{-\sum_{i}p_{n}(i)\log_{2}p_{n}(i)}{\log_{2}n}\in [0,1].$$

In the following we refer to the entropy of the probability density function as the normalized entropy of the probability density function, i.e., $H_{q}(f)\equiv NH_{q}(f)\in [0,1]$. 

### 2.3. Tail Entropy

Similarly to the definition of entropy, we can define tail entropy as being when entropy is computed using observations in the tail only. Shannon tail entropy is defined below.

**Definition** **6 (Shannon tail entropy).** *For X, a discrete random variable with probability distribution $X:\left(\begin{array}{l} x_{1}......x_{n}\\ p_{1}......p_{n} \end{array}\right)$, with $p_{i}=P(X=x_{i})$, $0\leq p_{i}\leq 1$, and $\sum_{i}p_{i}=1$, and α-level value-at-risk $VaR_{\alpha }\left(X\right)$ for 0 < α < 1, the Shannon tail information entropy at level α is defined by*

(15) $$H_{\alpha }(X)=-\sum_{x_{i}<VaR_{\alpha }\left(X\right)}p_{i}^{*}\log_{2}p_{i}^{*}.$$

The tail-adjusted probabilities are denoted by $p_{i}^{*}=p_{i}/\alpha$, and this normalization is required to make sure that the probabilities in the tail add up to 1. For a discrete distribution, the TE will reach its maximum value for a distribution which has uniform distribution in the α-tail, with all tail observations having the same probability. Similarly, for a given α, the tail entropy will reach its minimum for a distribution with zero uncertainty in the tail (an observation in the tail has a probability of p_i_ = α and the rest of the tail observations have a probability of 0).

For X, the discrete random variable given above, we can compute the expected shortfall based on the variable’s probability distribution at level α; this is denoted by ESPD and given by

(16) $$ESPD_{\alpha }(X)=\sum_{x_{i}<VaR_{\alpha }\left(X\right)}p_{i}^{*}x_{i}\;.$$

**Figure 1:**
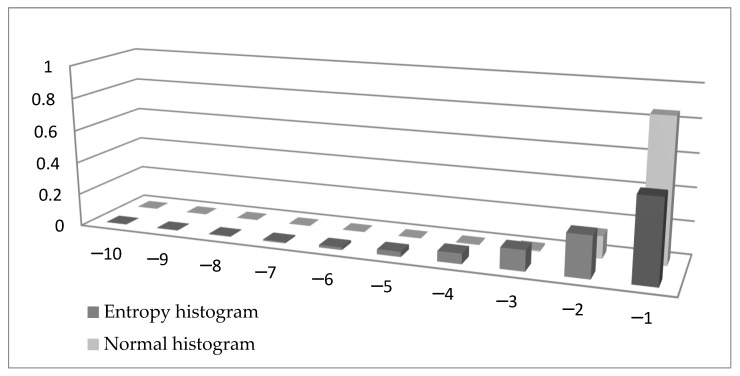
Comparison between a normal histogram and a histogram built using entropy.

The similarity between Formulas (15) and (16) is obvious. There is a minus sign in Formula (15) to make sure the tail entropy is a positive measure. More importantly, while the entropy is a weighted sum of log probabilities, the ES is the weighted sum of observations in the tail. To visualize the difference between the two formulae, in Figure 1 we compare the left tail of a histogram of a variable distributed N (0,1) (normalized with the tail probability and using bins of width 1) with the left tail of a histogram built using the entropy measure. In Formula (15) we can denote $\log_{2}p_{i}^{*}$ by *x_i_,* giving $p_{i}^{*}=2^{x_{i}}$, and this probability is presented in Figure 1 in the entropy histogram. It can be seen that the probabilities calculated using entropy are higher (except for when using the value −1) than the probabilities in the normal histogram.

*For a continuous random variable with density function*f(x), *the differential tail entropy is given by*

(17) $$H_{\alpha }(f)=-\int_{A}f(x)\log_{2}f(x)dx,\;A=\mathrm{supp}\left(X\right)\cap \left\{x<-VaR_{\alpha }\left(x\right)\right\}.$$

Similarly to differential entropy, tail differential entropy does not possess the properties of invariance to linear transformations and non-negativity. Using quantization, a measure of entropy similar to Shannon tail entropy can be defined as follows.

**Definition** **7 (tail entropy of a probability density function at quantization level *q*).** For the probability density function f:[0,1]→Im(f), let $I_{i}=[iq,(i+1)q)$ disjoint giving $\overset{n}{\underset{i=0}{\cup }}I_{i}=[0,1]$ and $B_{i}=f^{-1}(I_{i})$ not necessarily disjoint, with $\overset{n}{\underset{i=0}{\cup }}B_{i}=[0,1]$. Then, the tail entropy of f at level α with quantum q (using the notation μ* for the Lebesgue measure divided by α and [.] for the integer part of a number) is

(18) $$H_{\alpha ,q}(f)=-\sum_{i=0}^{\left[\frac{\alpha }{q}\right]}\mu^{*}(B_{i})\log_{2}(\mu^{*}(B_{i})).$$

Of note is that as in Definition 5, for a probability density function defined on set A which is not necessarily of finite measure, we can consider its restriction on a compact interval, i.e.,

(19) f|[a,b]:[a,b]→Im(f|[a,b]), f|[a,b](x)=f(x).

The tail entropy of the returns’ distribution can be interpreted as the expected shortfall of the returns, if in the tail of the distribution the probability of losses can be expressed as an exponential function of the size of the losses. The tail entropy is a measure of uncertainty in the tail, with low values of tail entropy being associated with a lower risk in the tail.

This framework can be used to estimate the tail entropy of a distribution function of a continuous random variable *X,* defined on the support set of *X*, with values on [0, 1]. The distribution function can be estimated using the histogram estimator of a probability density or kernel density estimation methods as described in the previous section. We proceed by presenting an estimation algorithm of the tail entropy of a distribution function.

Let $X_{0},....,X_{n-1}$ be a sample of an *i.i.d.* variable with probability density function *f*. The Algorithm 2 (see below) estimates the tail entropy of a distribution function.
**Algorithm 2. Estimation of the tail entropy of a distribution function.**Estimate the probability density function, obtaining values ${\hat{f}}_{n}(X_{i})$ for i=0,…n−1;Sample from the probability density function using the sampled function $S_{n}({\hat{f}}_{n})(i)={\hat{f}}_{n}(X_{i})$ for i=0,…n−1;Define a quantum q>0; then $Q_{q}S_{n}({\hat{f}}_{n})(j)=(i+1/2)q$, if ${\hat{f}}_{n}(X_{j})\in [iq,(i+1)q)$;Compute the probabilities $p_{n}^{*}(i)=\frac{c_{n}(i)}{\alpha \sum_{j}c_{n}(j)}=\frac{c_{n}(i)}{\alpha n}=\frac{card\{{\hat{f}}_{n}(X_{j})\in [iq,(i+1)q)\}}{\alpha n}$;Estimate the tail entropy of F as $H_{\alpha ,q}({\hat{f}}_{n})=-\sum_{i=0}^{m}p_{n}^{*}(i)\log_{2}p_{n}^{*}(i)$ where $m=\left[\frac{\alpha }{q}\right]$. 

The tail entropy of a distribution function reaches its maximum value for a distribution which has uniform distribution in the α-tail.

**Property** **(maximum value of the tail entropy of a probability density function):** *The α-tail entropy of a distribution function F:[0,1]→[0,1] reaches its maximum for a distribution with F(x) = x for x ≤ α, and this maximum value is $\log_{2}m$.*

**Proof.**  This is similar to the proof of the maximum Shannon entropy. Indeed, when all probabilities are equal for the observations in the α-tail (= 1/*m*), the tail probability can be written as:

(20) $$H_{\alpha ,q}({\hat{f}}_{n})=-\sum_{i=0}^{m}p_{n}^{*}(i)\log_{2}p_{n}^{*}(i)=-\sum_{i=0}^{m}q\log_{2}q=-\log_{2}q=\log_{2}m.$$

□

As such, a dimensionless measure of uncertainty, the normalized tail entropy, can be defined as the ratio between the entropy of the probability density function and its maximum value, i.e.,

(21) $$NH_{\alpha ,q}({\hat{f}}_{n})=\frac{-\sum_{i=0}^{m}p_{n}^{*}(i)\log_{2}p_{n}^{*}(i)}{\log_{2}m}\in [0,1].$$

We refer below to the tail entropy of the probability density function as the normalized tail entropy of the probability density function: $H_{\alpha ,q}(\hat{f})\equiv NH_{\alpha ,q}(\hat{f})\in [0,1]$.

In practical applications, the estimated tail entropy may be severely biased for small samples (Liu et al. [57]). In order to correct for small sample bias we propose a bootstrapping method, following DeDeo et al. [58]. Thus, we generate *B* independent samples of volume *k* from a multinomial distribution with probabilities $\hat{p}=(p_{n}^{*}(0),\ldots ,p_{n}^{*}(m))$, and for each sample we estimate the normalized tail entropy $H_{\alpha ,q}^{b}(\hat{f}),\;b=1\ldots B$. The unbiased estimator of the normalized tail entropy has the form

(22) $$H_{\alpha ,q}^{bootstrap}(\hat{f})=2H_{\alpha ,q}^{}(\hat{f})-\frac{1}{B}\sum_{b=1}^{B}H_{\alpha ,q}^{b}(\hat{f}).$$

The estimated entropy can be influenced by the quantum value *q >* 0; as there is no canonical quantum/level of quantization, apparently all results depend on an arbitrarily chosen quantum/quantization level. However, the Lorentz theorem ([54]) shows that the entropy estimator is consistent regardless of the choice of *q*; in practical applications, such as stock market data, we have used *q =* 0.2 (see Section 3). For *q =* 0.2, the number of bins used to compute the tail entropy is 1/*q* = 5; a smaller value of the quantum *q* will increase the number of bins and also the likelihood of having zero probability bins. For example, when the window used for estimation has 1000 observations (roughly 4 years of daily observations) and α = 5%, there are 50 observations in the left tail of the distribution; by decreasing the quantum level and increasing the number of bins, there is a high chance of having bins with zero probability where the term plogp is undefined.

Unlike expected shortfall, which is not well-defined when the expectation fails to exist (for instance for the Pareto family when the parameter *a* ≤ 1), tail entropy does exist for the Pareto distribution for any tail parameter value *a*. In general, under the hypothesis of the Lorentz theorem ([54]), tail entropy exists for any probability density function with bounded support. Even if the probability density function is not bounded, we can consider its restriction on a compact interval in order to fulfil the conditions of the Lorentz theorem. Indeed, tail entropy is sensitive to the choice of the level of the quantum, but in the next section we show how it can be transformed (via normalizing it and using a linear adjustment) into an expected-shortfall-type measure which will be less sensitive to the choice of quantum.

#### 2.3.1. Tail Entropy Expected Shortfall

Next, we turn our attention to the link between tail entropy and measures of risk and uncertainty, seeking to propose a tail-entropy-enhanced ES estimator. First, we perform a Monte Carlo experiment, estimating and comparing tail entropy and historical expected shortfall for several simulated Pareto-like distributions; we report our results for different tail probabilities. Here we use the random variable *Y* = −*X~Pareto*(*a*), which has the following characteristics:

(i) Cumulative distribution function: $F(x)=P(X<x)=1-{\left(\frac{c}{x}\right)}^{a},x>c>0$.
(ii) Probability density function: $f(x)=\frac{ac^{a}}{x^{a+1}},x>c$.
(iii) $P(X>x)={\left(\frac{c}{x}\right)}^{a}=Cx^{-a}$.

**Figure 2:**
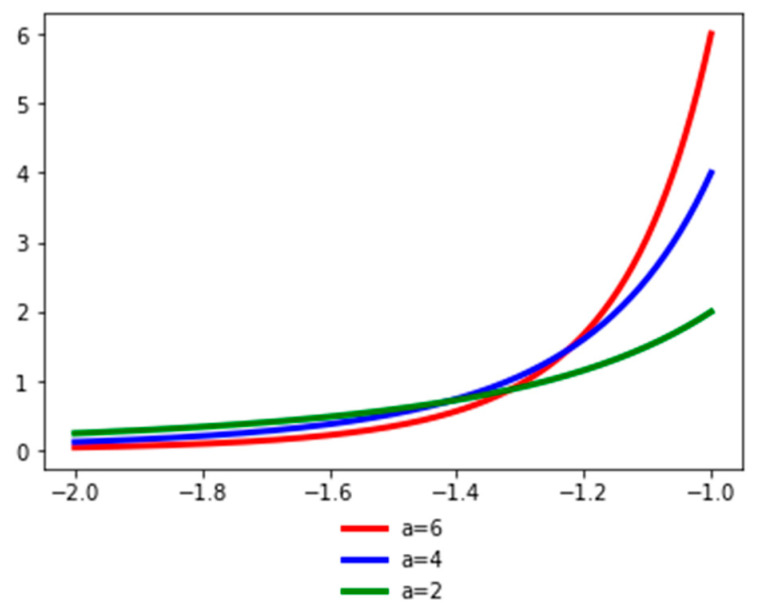
Probability density function for *Y*. See the Github page *SFM_Sim_Pareto_like*.

Figure 2 presents the probability density function of the random variable *Y* for different values of the *a* parameter. The *a* parameter (the tail index) is a measure of tail probability: higher values of *a* correspond to lower probability in the tail.

**Figure 3:**
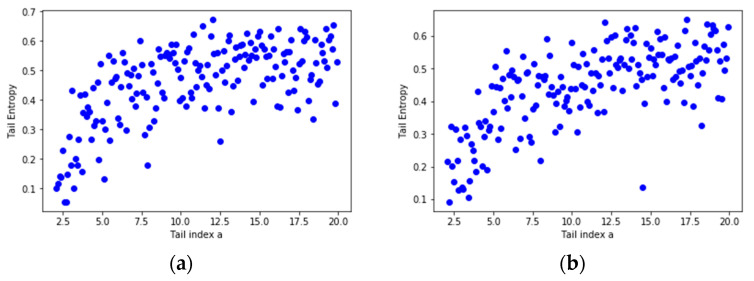
Tail Entropy and tail index *a* of the simulated distributions: (**a**) *α* = 1% and (**b**) *α* = 2.5%. See the Github page *SFM_ES_Pareto*.

**Figure 4:**
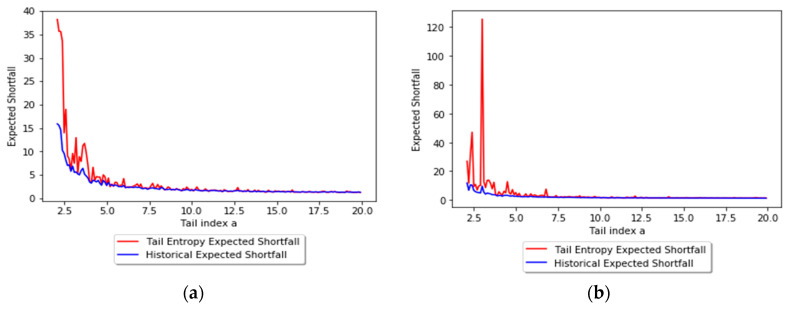
Tail entropy and historical expected shortfall of the simulated distributions: (**a**) *α* = 1% and (**b**) *α* = 2.5%. See the Github page *SFM_ES_Pareto*.

In order to assess the relationship between the tail entropy and the historical expected shortfall (ESH), we simulate random variables $Y_{k}=-X_{k}∼Pareto(a_{k})$, with $a_{k}=2+0.1k,\;k=1...180$. Figure 3 presents the relationship between TE and tail index *a* using scatterplots for different levels, i.e., 1% and 2.5%, of *α*. As the *a* parameter of the simulated distribution decreases, the tail entropy of the distribution decreases, too; as expected, high tail entropy values are associated with heavy-tailed distributions. Figure 4 presents the relationship between TE and historical ES, as functions of the *a* parameter of the simulated distribution, for 1% and 2.5% levels of *α*.

Additionally, we regress the historical ES estimates of the simulated returns above on tail entropy; the results are reported in Table 1. We estimate the historical ES using the formula

(23) $$ESH_{\alpha }\left(X\right)=-E\left[x|x\leq -VaR_{\alpha }\left(X\right)\right].$$

It can be seen that there is a strong linear negative relationship between the tail entropy and ESH, with higher values of tail entropy corresponding to lower values of expected shortfall. The validity of this regression approach depends on the tail behavior of the underlying distribution; without this explicit assumption, one would need regularly varying tails and a particular tail behavior in order to make the regression approach sound. Indeed, for some distributions the relationship between historical ES and tail entropy might not be exactly linear, but we show below how assuming a linear relationship helps us define a new measure of expected shortfall.

A multiple regression with several tail entropies of varying tail levels was also estimated in order to explicitly assess the relationship between historical ES and TE for a Pareto-like simulated distribution, as in the above examples.

Thus, we estimated the following regression model, where the historical ES (1% and 2.5%) is regressed against the tail entropy for significance levels 1%, 2.5%, and 5%, i.e.,

(24) $$ESH_{\alpha ;t}=\gamma_{0}+\gamma_{1}H_{0.01,q;t}+\gamma_{2}H_{0.025,q;t}+\gamma_{3}H_{0.05,q;t}+\eta_{t}.$$

The results of the regression as calculated using Formula (23) are reported in Table 2**.** By including varying tail levels for entropy, the performance of the model is significantly improved, with $R_{adj}^{2}$ increasing for α = 1% from 51% (see Table 1, left panel) to 55%, and for α = 2.5% from 48% (see Table 1, right panel) to 60%.

A second simulation using α-stable distributions can help understand this behavior. We chose α-stable distributions because they have several interesting properties: they allow for heavy tails and any linear combination of independent stable variables follows a stable distribution for up to a scale and location parameter (Nolan [59]); additionally, the Gaussian distribution is a particular case of a stable distribution.

We have that a random variable *X* which follows an α-stable distribution S(a,b,c,d;1) if its characteristic function is

(25) $$\varphi (t)=E[e^{itX}]=\left\{ \begin{array}{ll} \exp(-c^{a}{\left|t\right|}^{a}[1-ib\tan(\frac{\pi a}{2})sign(t)]+idt), & a\neq 1\\ \exp(-c\left|t\right|[1+ibt\frac{2}{\pi }sign(t)(\ln(\left|t\right|)]+idt), & a=1 \end{array}\right..$$

In the above expression a∈(0.2] is the tail index (we obtain a=2 for a normal distribution and lower values correspond to heavier tails), b∈[−1,1] is the skewness parameter, c∈(0,∞) is the scale parameter, and d∈R is the location parameter. We simulate 1000 observations from an α-stable distribution $S(a_{k},b,c,d;1)$ for $a_{k}=1.1+0.01k,\;k=1...90$ and study the relationship between tail index and tail entropy.

**Figure 5:**
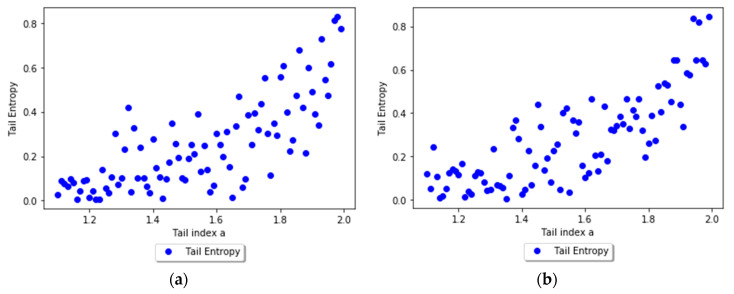
Tail Entropy and tail index of the simulated distributions: (**a**) *α* = 1% and (**b**) *α* = 2.5%. See the Github page *SFM_ES_Stable*.

The tail entropy reaches its maximum value for a distribution that has uniform distribution in the tail, and as the *a* parameter decreases, the tail entropy of the stable distribution decreases, too. As expected, low tail entropy values are associated with heavier-tailed distributions. Figure 5 presents the relationship between TE and tail index, while Table 3 present the regression results between TE and tail index.

Based on the results above (and taking into consideration that the tail entropy is between 0 and 1), we perform a linear adjustment on the tail entropy to obtain the tail entropy expected shortfall, i.e.,

(26) $$ESTE_{\alpha ,q}(f)=-\left[b_{0}+\left(\frac{b_{m}-b_{0}}{2}\right)H_{\alpha ,q}(f)\right],$$

where *b*_0_ is the mid-point of the first bin *B*_0_ and *b_m_* is the mid-point of the last bin in the α-tail of the distribution *B_m_*. 

The justification for Formula (25) lies in the following points.

- If the entropy reaches its maximum ($H_{\alpha ,q}(f)=1$), which corresponds to the highest uncertainty level, then we have $ESTE_{\alpha ,q}(f)=-\left[b_{0}+\left(\frac{b_{m}-b_{0}}{2}\right)\right]=-\left(\frac{b_{m}+b_{0}}{2}\right)$, i.e., the tail entropy expected shortfall is the average of losses below the value-at-risk.
- If the entropy reaches its minimum ($H_{\alpha ,q}(f)=0$), which corresponds to the lowest uncertainty level, then we have $ESTE_{\alpha ,q}(f)=-b_{0}$, i.e., the tail entropy expected shortfall is the minimum value of the distribution.

#### 2.3.2. Tail Entropy Expected Shortfall as a Spectral Risk Measure

According to Guégan et al. [8], a spectral risk measure can be defined as the function ρ:ℒ→R, where ℒ is a functional space and

(27) $$\rho (X)=-\int_{0}^{1}\phi (p)F^{-1}(x)(p)dp\;.$$

In Formula (26) ϕ is a positive or null, non-increasing, right-continuous, integrable function defined on [0, 1] such as $\int_{0}^{1}\phi (p)dp=1$.

Following Formula (25), the tail entropy expected shortfall can be written as a linear function of the tail entropy (even though for some distributions this is only an approximation), i.e.,

(28) $$ESTE_{\alpha ,q}(f)=-\left[X_{\min}+ES_{\alpha }(X)H_{\alpha ,q}(f)\right]=-\left[X_{\min}+\beta \int_{0}^{1}\phi (p)F^{-1}(x)(p)dp\right],$$

where $\beta =-\frac{1}{(4\ln2)\log_{2}m}=-\frac{1}{4\ln m}$ and $\phi (p)=-(4\ln2)p\log_{2}p=-4p\ln p$.

Thus, according to Formula (27), the tail entropy expected shortfall is a spectral risk measure and satisfies the conditions of coherence (see Artzner [1,2]), any spectral risk measure being law-invariant and comonotonic additive (see [8]).

The tail entropy expected shortfall can have interpretability in terms of monetary units, being a linear combination of the expected shortfall and the adimensional normalized entropy which takes values between [0,1], as can be seen from Formula (27).

#### 2.3.3. Backtesting ES

In order to forecast daily ES, non-parametric and parametric models are used based on a rolling window approach. Thus, we compare the ES forecasting ability of the following six models.

1. Historical ES forecasts: ${\hat{ESH}}_{\alpha ;k+w+1}^{}\left(X\right)=-E\left[x_{t}|x_{t}\leq -{\hat{VaR}}_{\alpha ;k+w+1}^{Historical}\right]$, where ${\hat{VaR}}_{\alpha ;k+w+1}^{Historical}$ is the α-quantile of the daily log-returns distribution;
2. Tail entropy ES forecasts: ${\hat{ESTE}}_{\alpha ,q;k+w+1}^{}(f)=-\left[b_{0}+\left(\frac{b_{m}-b_{0}}{2}\right)H_{\alpha ,q}(\hat{f})\right]$;
3. Normal GARCH(1,1) ES forecasts: ${\hat{ES}}_{\alpha ;k+w+1}^{N-GARCH(1,1)}\left(X\right)=-E\left[x_{t}|x_{t}\leq -{\hat{VaR}}_{\alpha ;k+w+1}^{N-GARCH(1,1)}\right]$, where ${\hat{VaR}}_{\alpha ;k+w+1}^{N-GARCH(1,1)}=-(z_{\alpha }{\hat{\sigma }}_{k+w+1}+\mu_{k+w+1})$, $z_{\alpha }$ being the Gaussian quantile;
4. Student’s *t* GARCH(1,1) ES forecasts: ${\hat{ES}}_{\alpha ;k+w+1}^{t-GARCH(1,1)}\left(X\right)=-E\left[x_{t}|x_{t}\leq -{\hat{VaR}}_{\alpha ;k+w+1}^{t-GARCH(1,1)}\right]$, where ${\hat{VaR}}_{\alpha ;k+w+1}^{t-GARCH(1,1)}=-(t_{\alpha ;df}{\hat{\sigma }}_{k+w+1}+\mu_{k+w+1})$, $t_{\alpha ;df}$ being the Student’s *t* quantile with *df* being the estimated degrees of freedom;
5. Gaussian ES forecasts: ${\hat{ES}}_{\alpha ;k+w+1}^{Gauss}\left(X\right)=-E\left[x_{t}|x_{t}\leq -{\hat{VaR}}_{\alpha ;k+w+1}^{Gauss}\right]$, where $-{\hat{VaR}}_{\alpha ;k+w+1}^{Gauss}$ is the α-quantile of the estimated Gaussian distribution of log-returns;
6. Student’s *t* ES forecasts: ${\hat{ES}}_{\alpha ;k+w+1}^{t}\left(X\right)=-E\left[x_{t}|x_{t}\leq -{\hat{VaR}}_{\alpha ;k+w+1}^{t}\right]$, where $-{\hat{VaR}}_{\alpha ;k+w+1}^{t}$ is the α-quantile of the estimated Student’s *t* distribution of log-returns.

In the above formulae, every risk measure is estimated using a rolling window of length *w*, with t∈{k+1,…,k+w}, k∈{0,…,T−w+1}, and *T* the number of daily log-returns. To see the ES forecasting performance of the above models, we run several backtests to compare the ES estimates. These are the unconditional exception frequency (UC) test and the conditional independence (CC) test of Du and Escanciano [60], and the $Z_{2}$ exception frequency and magnitude test of Acerbi and Szekely [61]. These are described in Appendix A and Appendix B. 

## 3. Empirical Analysis

**Figure 6:**
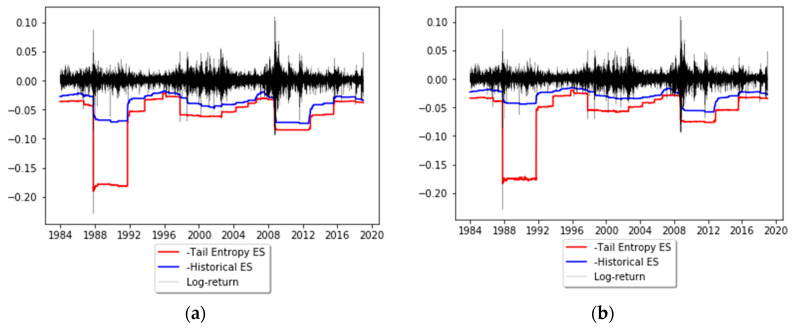
Dynamics of tail-entropy-based ES versus historical ES: (**a**) α = 1% and (**b**) α = 2.5%. See the Github page *SFM_TE_ES*.

In order to illustrate the application of tail entropy in financial risk management, we consider S&P500 daily log-returns (sourced from Bloomberg). The time period considered is January 1 1980 to December 12 2018 (9835 observations). The estimators of expected shortfall described in Section 2 are computed on a rolling basis using a length of *w* = 1000 days for the estimation windows; these forecasts are later subjected to ES backtests. Tail entropy is estimated using a quantum *q =* 0.2 (see the theorem in Section 2). Figure 6 depicts the dynamics of tail-entropy-based ES versus historical ES estimated at 1% and 2.5% significance levels. It can be noted that the tail-entropy-based ES gives higher estimates of risk compared to the historical ES for both significance levels, as this model assigns a higher probability to large losses when compared to the historical ES model. However, it is not straightforward to comment on the correctness of these risk estimates as the level of true risk is not observable, meaning no direct comparisons can be made for true risk. Statistical backtests, performed in the next section, can be used to verify the correctness of VaR and ES forecasts.

### Backtesting ES

Backtesting of the ES forecasts based on the models given in Section 2.3 was performed using a window length of 1000 trading days and for α = 1% and 2.5%. Table 4 presents the percentage of times each model passed the given backtest for each significance level α. Based on these statistics, it can be concluded that for the unconditional ES test, the *t*-GARCH(1,1) ES forecasts perform best for both levels of α. Looking at the rejection rates for the conditional coverage ES backtest, it can be seen that at the 2.5% level the *t*-GARCH(1,1) model performs best but at the 1% level the rejection rate of the tail entropy ES is comparable with the same rate within the *t*-GARCH(1,1) model. Also, according to the $Z_{2}$ test, the rejection rates are the lowest for tail entropy ES for α = 2.5%, and stand at around 10%. Based on these results the *t*-GARCH(1,1) and tail entropy ES show similar performances and overperform the other models considered. Table 5 presents the test statistics of each backtest over the entire testing period and for α levels of 1% and 2.5%. The tail entropy ES, like the other ES models considered, fails both the unconditional and conditional backtests at the 5% level, but it passes the $Z_{2}$ test at the 5% level, which the *t*-GARCH(1,1) ES forecasts fail to pass.

**Figure 7:**
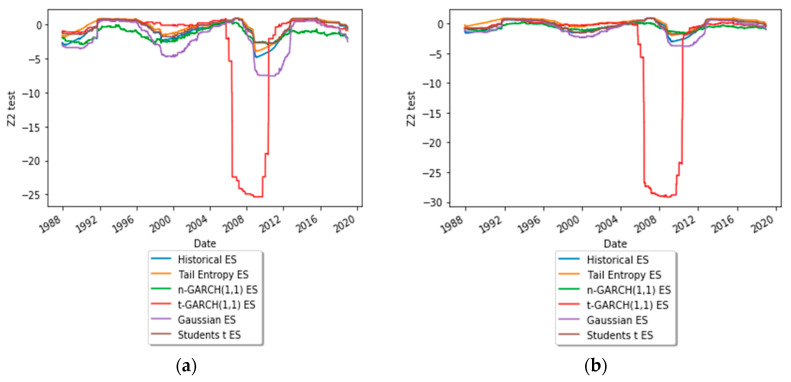
Dynamics of the $Z_{2}$ test statistic: (**a**) α = 1% and (**b**) α = 2.5%. See the Github page *SFM_TE_ES*.

To get an insight into the dynamic behavior of the test statistics of the $Z_{2}$ backtest, Figure 7 depicts time-varying test statistics (using a rolling window of length 1000) for ES estimated at significance levels α = 1% and 2.5%. As can be seen, the expected shortfall estimated with the Student’s *t*-GARCH(1,1) model underperformed, for both α levels, during the financial crisis of 2008.

The illustration of tail-Entropy-based ES in Figure 6 indicates that at similar backtesting rejection rates tail-entropy-based ES seems to lead to overly high risk quantifications; in addition, the time to recover from a bad shock (like Black Monday on 19 October 1987) is much longer than for historical expected shortfall (which itself usually leads to long recovery times). These two observations seem to indicate that the proposed entropy-based version of expected shortfall tends to react fiercely in the presence of outlying observations. This may be a sign that tail-entropy-based ES is a more conservative risk measure than traditional ones due to the way it is defined, as it takes into account the tail distribution, which has a higher inertia and a longer recovery time from a negative shock.

## 4. Conclusions

In this paper we propose a nonparametric estimator of ES based on TE, as well as an extension of this model based on kernel smoothing, and compare it with the classical estimates of ES, i.e., historical ES, Gaussian ES, Student’s *t* ES, Gaussian GARCH(1,1) ES and Student’s *t*-GARCH(1,1) ES. The main advantage of the measure we propose is that it has a low dependency on the actual values of observations in the tail of the distribution, making it a more stable measure of tail risk.

We illustrate an application of tail entropy in financial risk management based on S&P 500 daily log-returns between 1980 and 2018 (9835 observations). Comparing backtest rejection rates based on rolling windows, it can be concluded that the performance of tail entropy ES is comparable with the performance of the *t*-GARCH(1,1) model. However, when we backtest over the entire sample period, tail-entropy-based ES is the only model which passed the $Z_{2}$ backtest.

Further research is needed to fully acknowledge the efficiency of the tail entropy ES estimator. This new measure may be beneficial for risk measurement as it is fully non-parametric and has a reduced sensitivity to actual observations in the tail. One possible extension of this approach will be to assess the predictive performance of tail entropy ES in a non-standard environment, for example by assuming a fat-tailed distribution of the returns as the generalized error distribution (see Cerqueti et al., 2019 [62]). In addition, our approach can be applied to other fields such as operational risk, where distributions are usually fat-tailed and highly skewed.

Our research can be easily reproduced using the Python codes uploaded to www.github.com.

## Figures and Tables

**Table 1:** Relationship between historical ES (ESH) and tail entropy (TE) for simulated distributions.

Dependent Variable: *ESH*_α;*t*_		
Significance Level	α = 1%	α = 2.5%
*H_α,q;t_*	−12.95 ***	−8.32 ***
	[0.94]	[0.64]
$R_{adj}^{2}$	0.51	0.48

Note: standard errors are given in brackets. *** denotes significance at 1%.

**Table 2:** The results of the multivariate regression model obtained using Formula (23).

Dependent Variable: *ESH*_α;*t*_		
Significance Level	α = 1%	α = 2.5%
*Intercept*	5.75 ***	4.32 ***
	[0.13]	[0.08]
*H_0.01,q;t_*	−3.69 ***	−2.48 ***
	[0.37]	[0.23]
*H_0.025,q;t_*	−3.64 ***	−2.57 ***
	[0.38]	[0.24]
*H_0.05,q;t_*	−3.07 ***	−2.20 ***
	[0.37]	[0.23]
$R_{adj}^{2}$	0.55	0.60

Note: standard errors are given in brackets. *** denotes significance at 1%.

**Table 3:** Relationship between tail index and TE of simulated distributions.

Dependent Variable: *TE*_α;*t*_		
Significance Level	α = 1%	α = 2.5%
*a*	0.51 ***	0.63 ***
	[0.05]	[0.05]
$R_{adj}^{2}$	0.50	0.65

Note: standard errors are given in brackets. *** denotes significance at 1%.

**Table 4:** Backtesting rejection rates of the null hypothesis from January 1980 to December 2018. Legend: UC, unconditional exception frequency; CC, conditional independence.

	α = 1%			α = 2.5%		
Model	Du’s UC Test	Du’s CC Test	$Z_{2}\;\mathrm{Test}$	Du’s UC Test	Du’s CC Test	$Z_{2}\;\mathrm{Test}$
Gaussian ES	62.56%	56.43%	55.55%	74.74%	59.17%	43.93%
Students’s *t* ES	65.93%	42.80%	38.04%	73.41%	66.03%	34.35%
Historical ES	59.02%	38.14%	39.82%	73.39%	69.79%	33.00%
n-GARCH(1,1) ES	74.83%	33.05%	77.03%	66.91%	46.57%	45.77%
*t*-GARCH(1,1) ES	29.30%	23.17%	26.73%	27.44%	41.97%	23.32%
Tail Entropy ES	58.35%	24.11%	28.29%	69.50%	69.22%	10.25%

Note: rolling windows of length 1000 were used to carry out the backtests.

**Table 5:** Test statistics of ES backtests from January 1980 to December 2018.

	α = 1%			α = 2.5%		
Model	Du’s UC Test	Du’s CC Test	$Z_{2}\;\mathrm{Test}$	Du’s UC Test	Du’s CC Test	$Z_{2}\;\mathrm{Test}$
Gaussian ES	18.26	97.55	−1.88	11.18	84.61	−0.73
Student’s *t* ES	5.24	45.58	−0.33 *	5.48	74.74	−0.19 *
Historical ES	5.50	44.70	−0.62 *	4.56	81.62	−0.32 *
n-GARCH(1,1) ES	11.43	22.88	−1.39	7.78	18.39	−0.73
*t*-GARCH(1,1) ES	2.82	16.21	−2.78	3.39	22.37	−3.37
Tail Entropy ES	6.11	20.58	−0.23 *	20.71	66.28	0.20 *

Note: critical values for each test are the following. Du’s UC test (5%, two-sided, normal): −1.96, 1.96. Du’s CC test (5%, one-sided, Chi-squared): 3.84. $Z_{2}$ test (5%, one-sided, normal): −0.70. * stands for test statistics that passed the backtests at the 5% level.

## Appendix A. Unconditional and Conditional Backtests for ES (Du and Escanciano)

Let *x_t_*, *t* = 1 to *T* and denote the returns series, which are assumed to follow a model with estimated cdf ${\hat{F}}_{t}$ and estimated parameters ${\hat{\theta }}_{1}$. Denoting by Ω*_t_* the information available at time *t*, we define the cumulative probability under the estimated distribution and the cumulative violations, i.e.,

(A1) $${\hat{u}}_{t}={\hat{F}}_{t}\left(x_{t},\Omega_{t-1},{\hat{\theta }}_{1}\right),$$

(A2) $${\hat{H}}_{t}\left(\alpha \right)=\frac{1}{\alpha }\left(\alpha -{\hat{u}}_{t}\right)1_{{\hat{u}}_{t}\leq \alpha }.$$

The null hypothesis for the unconditional backtest is

(A3) $$H_{0U}:E\left(H_{t}\left(\alpha ,\theta_{0}\right)-\alpha /2\right)=0.$$

If the estimation period is much larger than the evaluation period, the unconditional test statistic and its distribution will be

(A4) $$U_{ES}=\frac{\sqrt{n}\left(\frac{1}{n}\sum_{t=1}^{n}{\hat{H}}_{t}\left(\alpha \right)-\frac{\alpha }{2}\right)}{\sqrt{\alpha \left(1/3-\alpha /4\right)}}\overset{d}{\to }N\left(0,1\right).$$

For the conditional backtest the null hypothesis is:

(A5) $$H_{0C}:E\left(H_{t}\left(\alpha ,\theta_{0}\right)-\alpha /2|\Omega_{t-1}\right)=0.$$

The conditional test statistic (for first order dependence) and its distribution is

(A6) $$C_{ES}\left(1\right)=\frac{n^{3}}{{\left(n-1\right)}^{2}}\cdot {\left(\frac{\sum_{t=2}^{n}\left({\hat{H}}_{t}\left(\alpha \right)-\alpha /2\right)\left({\hat{H}}_{t-1}\left(\alpha \right)-\alpha /2\right)}{\sum_{t=1}^{n}{\left({\hat{H}}_{t}\left(\alpha \right)-\alpha /2\right)}^{2}}\right)}^{2}\overset{d}{\to }\chi^{2}\left(1\right).$$

## Appendix B. Exception Frequency and Magnitude Test for ES (Acerbi and Szekely)

Let *x_t_*, *t* = 1 to *T* and denote the returns series with unknown tail distribution cdf $F_{t}$. When forecasting the risk of the returns, namely $VaR_{\alpha ,t}$ and $ES_{\alpha ,t}$, we obtain a predictive tail distribution $P_{t}$.

The hypotheses to be tested are

$H_{0}:P_{t}^{\left[\alpha \right]}=F_{t}^{\left[\alpha \right]}$

$H_{1}:ES_{\alpha ,t}^{F}\geq ES_{\alpha ,t}^{}$ for all *t* and > for some *t*

$VaR_{\alpha ,t}^{F}\geq VaR_{\alpha ,t}^{}$, for all *t*.

The test statistic is computed as

(A7) $$Z_{2}=\sum_{t=1}^{T}\frac{x_{t}1_{x_{t}<VaR_{\alpha ,t}}}{T\alpha ES_{\alpha ,t}}+1\;.$$

Under the null hypothesis we have $E_{H_{0}}[Z_{2}]=0$ and $E_{H_{1}}[Z_{2}]<0$. If the test statistic is below −0.7 then the null hypothesis is rejected and the model fails the ES backtest.
